# Supplementary material for: Nationwide Screening Practices for Tamoxifen Retinal Toxicity in South Korea: A Population-Based Cohort Study
Source: J Clin Med. 2024 Apr 9;13(8):2167. doi: 10.3390/jcm13082167 (PMC11050852; doi:10.3390/jcm13082167)
Supplement: Supplementary file 1 [file jcm-13-02167-s001.zip › jcm-2897510-supplementary.pdf]

Supplementary Figure

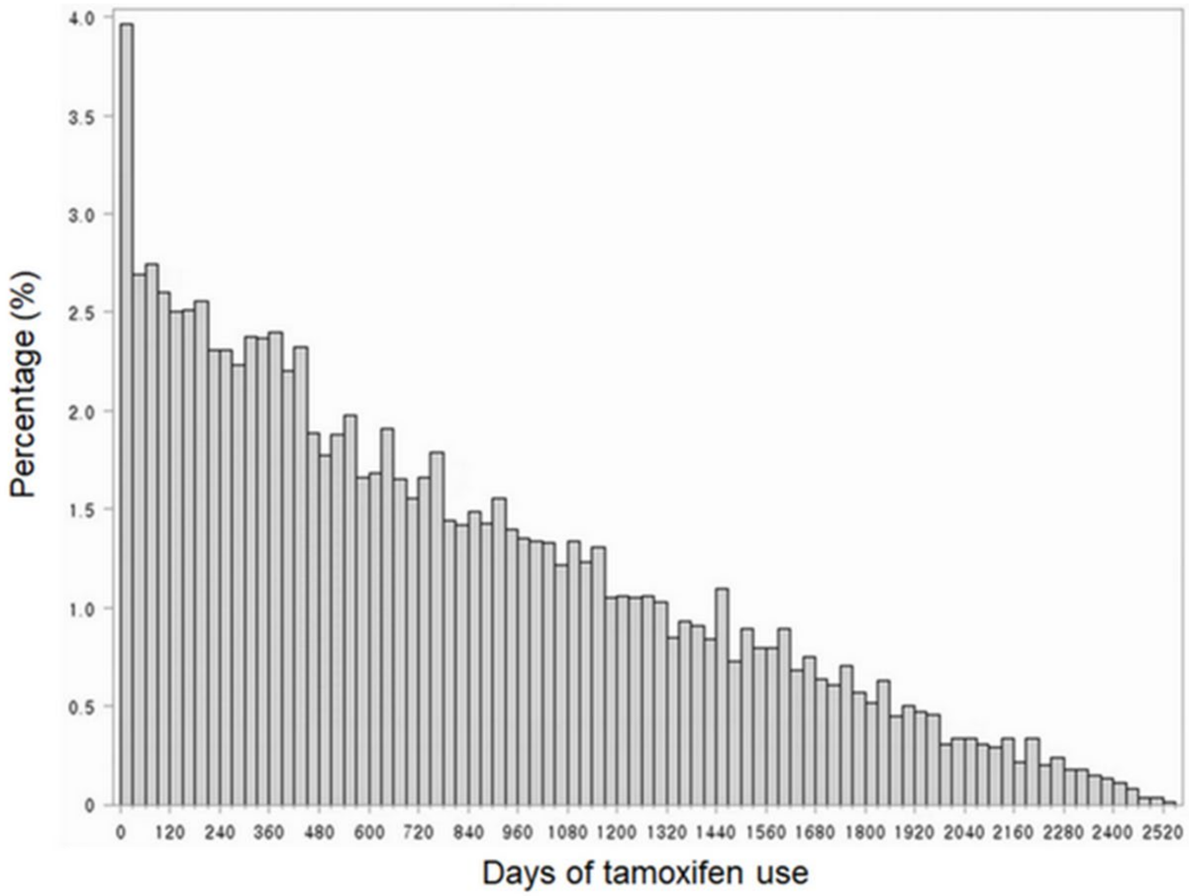

Supplementary Figure S1. Timing of Baseline Screening for Tamoxifen Retinopathy. The figure illustrates the timing of baseline screening for tamoxifen retinopathy, presented as the interval from the start date of tamoxifen treatment.

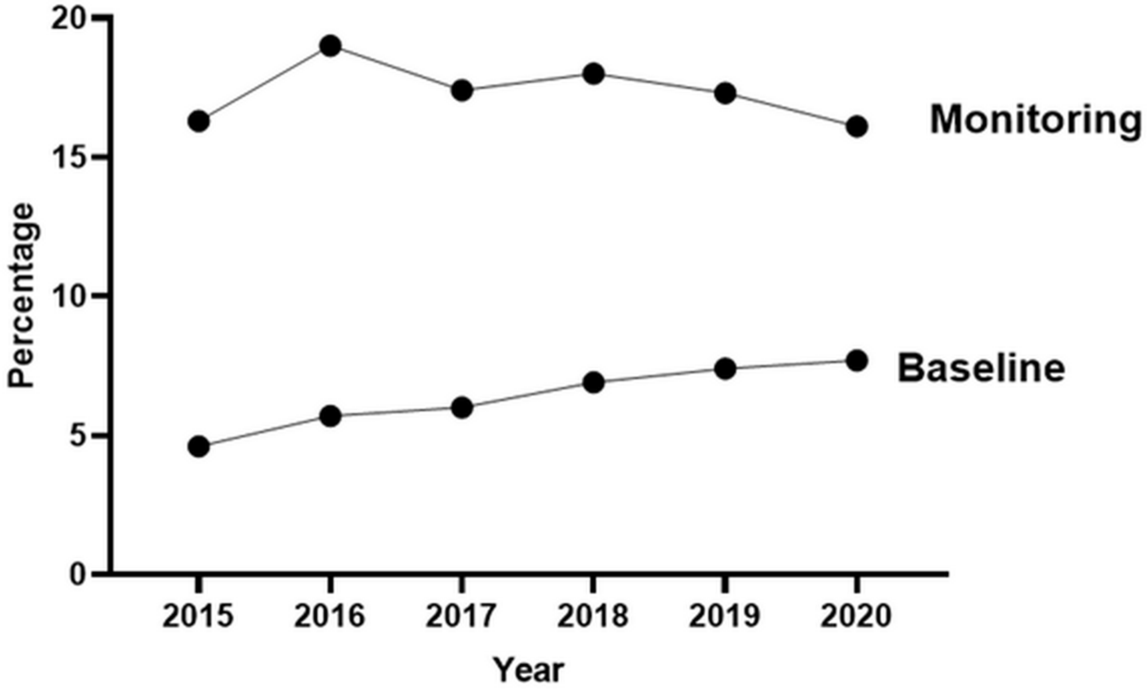

Supplementary Figure S2. Proportion of patients receiving baseline screening within 1 year of tamoxifen use and that of those with subsequent monitoring (within 1 year from the baseline examination) among those with baseline screening in each year between 2015 and 2020
